# The adenoviral E4orf4 protein: A multifunctional protein serving as a guide for treating cancer, a multifactorial disease

**DOI:** 10.1016/j.tvr.2024.200303

**Published:** 2024-12-15

**Authors:** Amir Basis, Rakefet Sharf, Tamar Kleinberger

**Affiliations:** Dept. of Molecular Microbiology, The Rappaport Faculty of Medicine and Research Institute, Technion-Israel Institute of Technology, Haifa, Israel

**Keywords:** Adenovirus, E4orf4, Virus-host interactions, Cancer therapy, Drug cocktail

## Abstract

Viruses exploit several cellular pathways to support their replication, and many of these virus-targeted pathways are also important for cancer growth. Consequently, studying virus-host interactions offers valuable insights into tumorigenesis and can suggest the development of novel anti-cancer therapies, with oncolytic viruses being one well-known example. The adenovirus E4orf4 protein, which disrupts several host regulatory pathways to facilitate viral infection, also functions as a potent anti-cancer agent when expressed independently. E4orf4 can selectively kill a wide range of cancer cell lines while sparing non-cancerous cells. Moreover, it effectively eliminated cancer in an *in vivo Drosophila* model without causing significant harm to normal tissues.

In this study we provide evidence that an E4orf4-mimicking drug cocktail, comprising sublethal doses of four FDA-approved drugs targeting the pathways disrupted by E4orf4, significantly enhanced cancer cell death in many cancer cell types compared with individual drugs or less inclusive drug combinations. The quadruple drug cocktail was not toxic in non-cancerous cells. These findings provide a proof-of-principle for the potential application of virus-host interaction studies to design an effective E4orf4-based cancer therapy. Further investigation of E4orf4 interactions with the host cell will likely improve this E4orf4-based therapy by adding drugs that disrupt additional pathways.

Crucially, the E4orf4-based approach offers a strategic advantage by avoiding the time-consuming development of novel drugs. Instead, it leverages existing drugs, including those that might be too toxic for use as monotherapies, by employing them at sublethal concentrations in combination. Thus, it provides a feasible and efficient method for advancing cancer therapy.

## 1 Introduction

Cancer has plagued humanity for millennia, with historical records dating back to ancient times, documenting its impact on human health [1]. In recent years, the global prevalence of cancer has increased significantly, driven by factors such as environmental influences, lifestyle choices, and the increasing average human lifespan [2]. This rise in cancer cases was accompanied by significant advances in our understanding of the disease, which have come a long way since the early days of cancer therapy. Initial notable milestones included Bernard Peyrilhe's first radical mastectomy for breast cancer in 1773 [3], the pioneering use of radiation therapy by Swedish physicians Tor Stenbeck and Tage Sjogren to treat squamous cell carcinoma in 1899 [4], and Roy Hertz and Min Chiu Li's groundbreaking use of methotrexate to achieve the first complete cure of a human solid tumor in 1953 [5]. However, the progress in developing novel cancer therapies has greatly accelerated since then.

With advancements in technology and scientific knowledge, we now have a deeper understanding of the genetic, environmental, physiological, and metabolic factors contributing to cancer initiation and progression. Despite these advances, one of the primary challenges in cancer treatment remains the ability to specifically target cancer cells without harming healthy tissues. This has led to an intense focus within the scientific community on developing new effective therapies, often in the form of targeted treatments by single agents.

One such prominent example is the introduction of monoclonal antibodies designed to target either cancer- or immune-expressed antigens, thus facilitating drug delivery or immune modulation respectively [6]. Another promising approach is the use of oncolytic viruses, which exploit the natural ability of wild type or mutant viruses to selectively infect and destroy cancer cells or deliver therapeutic genes to specific cells based on the virus's natural or modified tropism [7]. Additionally, drugs that target specific proteins altered in cancer cells, such as protein kinases, have shown significant promise in treating various types of cancer [8,9].

While these new therapeutic developments hold great promise and are already being applied in clinical practice, they face two fundamental challenges. The first challenge is identifying antigens or receptors that are uniquely or predominantly expressed in cancer cells, to ensure precise targeting. The second challenge is that many of these treatments focus on a single tumorigenic pathway. Given the complex and multifactorial nature of cancer, which involves mechanisms such as evading apoptosis, altering cellular metabolism, escaping immune detection, and maintaining unlimited replicative potential, a more comprehensive approach is needed. Furthermore, cancer cells are highly adaptive, often accumulating mutations that lead to drug resistance [10,11]. Therefore, targeting multiple tumorigenic pathways simultaneously makes more biological sense.

To address these challenges and overcome drug resistance, combination therapy is increasingly becoming the standard of care in many cancers where single-agent therapies are ineffective [12]. Combination therapies have the potential to target multiple pathways simultaneously, increasing treatment efficacy while potentially reducing side effects by allowing for lower doses of individual drugs [12,13]. However, discovering effective drug combinations that eliminate cancer cells while minimizing toxicity to normal cells poses a significant obstacle, as the number of possible combinations is too vast to be empirically tested [14].

In this manuscript, we present a novel approach to addressing the challenges facing cancer therapy by leveraging insights gained from studying virus-host interactions for the design of anti-cancer drug cocktails.

The adenoviral (Ad) protein E4 open reading frame 4 (E4orf4) is one of seven proteins encoded by alternatively-spliced RNAs transcribed from the early viral gene E4, which is activated by the Ad early region 1A (E1A) transcriptional activator early during infection [15]. E4orf4 is a small 14-kDa polypeptide containing 114 amino acid residues with no known structure other than that suggested by in-silico predicted models [16,17]. Research focusing on E4orf4 alone, or utilizing viruses lacking the rest of the E4 gene region, has revealed many functions of E4orf4 that enhance the efficiency of virus infection. These functions depend on the interactions of E4orf4 with various cellular protein partners. Some E4orf4 activities supplement the functions of other Ad proteins, such as inhibition of the DNA damage response (DDR, see below). In other cases, E4orf4 can function as a viral "rheostat", modulating the outcome of the activities of other Ad proteins. For example, once accumulated in the cells, E4orf4 reduces E1A-induced early Ad gene expression [18]. This E4orf4 function facilitates an efficient progression from the early to the intermediate and late stages of Ad infection and helps maintain a balance between high levels of virus replication and premature cell death [18].

The E4orf4 signaling network has a high degree of complexity as E4orf4 associates with several cellular proteins and through these interactions targets numerous regulatory pathways [18,19].

One signaling network targeted by E4orf4 is the DDR. Recent publications indicate that E4orf4 plays a role in the virus's efforts to inhibit the DDR, a regulatory network that serves as an antiviral defense system [[20], [21], [22]]. This E4orf4 function was investigated using a mutant virus lacking the other E4orf proteins, allowing selective and isolated examination of E4orf4 effects on the DDR. While a virus lacking the entire E4 gene region led to a robust activation of ataxia-telangiectasia mutated (ATM) and ATM and Rad3 related (ATR) signaling, the re-introduction of E4orf4 into the same virus significantly diminished the phosphorylation of DDR proteins and consequently their activation. When E4orf4 was expressed alone, it inhibited drug-induced DDR activation and DNA repair, ultimately resulting in an increased sensitivity of the cells to DNA damage-induced toxicity. Thus, DDR inhibition contributed to E4orf4-induced cell death manifested outside the context of virus infection [21]. Further investigation of the underlying mechanism revealed that E4orf4 interacted with two DNA damage sensors, poly (ADP-ribose) polymerase 1 (PARP-1) and DNA-dependent protein kinase (DNA-PK) [20,22]. The results indicated that E4orf4 was recruited to DNA damage sites in a PARP-1- and DNA-PK-dependent process, subsequently inhibiting these enzymes and blocking their DNA damage signaling cascades. During virus infection, inhibition of PARP-1 and DNA-PK increased replication efficiency [[20], [21], [22]].

Another example of cellular pathways targeted by E4orf4 is the cell cycle regulation network. In general, DNA virus infections often lead to modulation of the cell cycle, allowing a more suitable cellular environment for viral replication. During Ad infection, several viral proteins play a role in this process. For example, the Ad E1A protein stimulates progression of non-proliferating infected cells into S-phase thus facilitating the provision of factors required for virus DNA replication such as dNTPs [23]. The effects of E4orf4 on cell cycle regulation have been investigated in two biological systems, mammalian cells and the yeast *Saccharomyces cerevisiae*. Studies showed that E4orf4 caused an irreversible growth arrest at the G2/M phase of the cell cycle in yeast. This effect was mediated through E4orf4's interaction with subunit B55 of protein phosphatase 2A (PP2A), known in *S.cerevisiae* as cell division cycle 55 (Cdc55) [24,25]. Imaging of spindles using a GFP-Tubulin fluorescent protein showed that the majority of cells expressing E4orf4 were arrested either at pre-anaphase, marked by short spindles, or at telophase, marked by elongated spindles. Additionally, it was demonstrated that E4orf4 interacted with the anaphase-promoting complex/cyclosome (APC/C), a key spindle checkpoint complex that functions as an ubiquitin ligase responsible for degradation of cell cycle regulators. E4orf4 caused increased stability and accumulation of the APC/C substrates Pds1 and Ase1, demonstrating inhibition of APC/C complexes. Similarly, expression of E4orf4 in HEK293-derived cell lines led to an accumulation of cells with 4N DNA content, characteristic of cells in the G2/M phase. This population of E4orf4-expressing cells subsequently progressed to apoptosis [24]. In some cell lines the cells with 4N DNA content progressed to the G1 phase without going through cytokinesis and caused mitotic catastrophe [26,27]. It has been proposed that a G2/M arrest may prolong the replicative proficiency of the cells, thus supporting DNA virus replication under conditions where infection has occurred at late S-phase or when there is a deficiency in viral proteins regulating entry into S phase, such as the Ad E1A protein. It has indeed been demonstrated that the induction of cell cycle arrest at G2/M occurred during infection by various viruses, indicating that it may provide benefits for virus replication [28].

An additional pathway targeted by Ad is the phosphoinositide 3-kinase (PI3K) pathway. This pathway is normally activated by insulin or other growth factors and regulates several cellular processes including cell growth, proliferation, and metabolism [29]**.** PI3K signaling is activated in many cancers, which require enhanced glycolysis, a downstream target of PI3K [30,31]**.** As infection by many viruses also requires enhanced glycolysis to allow efficient viral replication, several viruses, including Ads, stimulate PI3K signaling. A major Ad protein that enhances glycolysis is E4orf1, which activates PI3K signaling [32,33] and improves expression of glycolytic proteins through its interaction with the Myc transcription activator [34]. Recent work from our laboratory demonstrates that E4orf4 counteracts PI3K-induced stimulation of glycolysis because excessive glycolysis is not beneficial for the virus (Agbaria et al., manuscript in preparation).

The mammalian target of rapamycin (mTOR) is another target of the PI3K pathway, and previous work has shown that E4orf4 activates mTOR in a PI3K-independent manner and that this activation is required for assembly of protein translation complexes and DNA synthesis [33]. Thus, while on the one hand, E4orf4 reduces PI3K signaling to avoid excessive glycolysis, it maintains activation of another PI3K target, mTOR, which is required for other processes supporting virus replication.

When expressed alone, outside the context of virus infection, E4orf4 induces cancer-selective cell death with unique features. Early studies showed that expressing E4orf4 alone in various transformed cell lines, including those lacking wild-type p53 expression, caused morphological changes indicative of cell death. These changes included early membrane blebbing, cell rounding, and the presence of condensed and fragmented nuclei typical of apoptosis [[35], [36], [37]]. Additional studies of E4orf4-induced cell death confirmed that E4orf4 was lethal to several human cancer cell lines from different cancer types but did not affect normal cells [38,39]. Moreover, *in vivo* research using a *Drosophila* cancer model found that E4orf4 not only inhibited tumor formation but also improved fly survival, even when highly aggressive and metastatic tumors were induced [40]. In contrast, E4orf4 had only a mild effect on tissue morphology when expressed in normal flies; it inhibited classical apoptosis in normal tissues, and had no effect on normal fly survival [41]. These results suggest that E4orf4 could be a potent anticancer agent with minimal toxicity in normal tissues, and offer a strong rationale for developing cancer treatments based on this protein.

In light of the above, taking into consideration that cancer is often driven by alterations in many cellular pathways, it makes sense that the multi functionality of E4orf4 and its ability to target regulatory pathways that are also required for cancer cell proliferation and maintenance, grants it the edge needed to disrupt cancer progression and proliferation. E4orf4's potency in cancer cells can be attributed at least in part to the high degree of its signaling network complexity, affecting several cellular pathways simultaneously. However, using the E4orf4 protein itself as a treatment for cancer is still unfeasible to date and imposes major challenges including protein purification, protein delivery into cells, targeting, immunogenicity, as well as many other pharmacokinetics and dynamics aspects.

Nevertheless, based on studies of the interactions between E4orf4 and each of its target pathways, we suggest that it is possible to achieve E4orf4-mimicking effects using several in-market drugs designed to target these same pathways individually. Moreover, we predicted that by utilizing numerous E4orf4-mimicking drugs simultaneously it would be possible to employ sublethal concentrations of each drug to obtain the full E4orf4-like effect and reduce toxicity in normal cells. We show here results supporting our hypothesis, confirming that deciphering the details of virus-host interactions is not only important for understanding virus biology but can also provide new approaches for cancer therapy.

## 2 Materials and methods

### 2.1 Cell culture

A549 lung cancer, HCT116 colon cancer, MDA-MB-231 breast cancer and HaCaT immortalized human keratinocytes cell lines were grown in DMEM medium with 10 % fetal bovine serum supplemented with glutamine and penicillin/streptomycin, in a 37C, 5 % CO2 incubator according to standard practice. HaCaT cells represent non-cancerous cell lines. PC3 prostate cancer cells were grown similarly with F12 medium instead of DMEM. All experiments were performed within a maximum of 10 passages after thawing cells from liquid nitrogen.

### 2.2 Assays of E4orf4-induced cell death

A DAPI assay was used to assess E4orf4-induced cell death, as described previously [42,43]. Briefly, the cells were transfected with a control plasmid expressing RFP or a parallel E4orf4-expressing plasmid. Forty-eight hours later, the cells were fixed with 4 % paraformaldehyde and E4orf4-expressing cells were stained with an E4orf4-specific antibody. All cells were counterstained with DAPI (4′,6′-diamidino-2- phenylindole, Sigma). Fluorescent cells were visualized by a Zeiss LSM 700 confocal microscope at ×40 magnification, using Imaris Viewer for analysis. The fraction of transfected cells expressing either control RFP or E4orf4, which manifested condensed nuclei, was determined in each experiment by counting at least 120 transfected nuclei.

A clonogenic assay was utilized for assessment of long-term survival of control and E4orf4-expressing cells. The cells were transfected with an empty vector, pcDNA4TO, or with pcDNA4TO-E4orf4. These plasmids express a zeocin selection marker. Cells were harvested two days post-transfection and were plated in six-well plates at a density of 5,000 or 10,000 cells per well. Another portion of the cells was subjected to Western blot analysis for validation of E4orf4 expression. Selection with zeocin was carried out for 10 days. The emergent colonies were then rinsed with PBS, fixed with methanol/acetic acid (3:1) for 30 min, stained in a solution of 1 % crystal violet (Sigma) in H2O, and colony number was scored.

### 2.3 Determination of sublethal drug doses

The drugs utilized here were: olaparib (Thermo Scientific, Cat no. 466292500), idelalisib (Cayman Chemicals, Cat no. 15279), paclitaxel (Cayman Chemicals, Cat no. 10461), and bleomycin (Abcam, Cat no. ab142977). All stock solutions were prepared in DMSO and stored at −20C. To establish sublethal concentrations of individual drugs, cells were seeded in a 96-well plate 24 h prior to treatment, at a density of 1,500 cells per well. The cells were then incubated in medium containing the desired drug concentrations for 48 h, and were then examined using the Sulforhodamine B based *in-vitro* assay for cytotoxicity, according to the manufacturer's specifications (Sigma, TOX6). Absorbance readings were performed using the TECAN Infinite® 200 PRO plate reader. Sublethal concentrations of drugs in each cell line were defined as concentrations that caused a 10 % or less decrease of growth in comparison to control untreated cells. DMSO concentrations did not exceed 0.2 % in all experiments.

### 2.4 Determination of the toxicity of drug combinations

For E4orf4-emulating drug combination treatments, cells were seeded at a density of 1,500 cells per well 24 h prior to treatment initiation. The cells were then subjected to individual drug treatment as well as to various drug combinations. Medium and drugs were refreshed at day 3 post-seeding and cells were assessed for toxicity at the end of day 4 post-seeding, using the sulforhodamine B assay, as described above. In this protocol, drug exposure lasted for 3.5 days. When an alamar blue cell viability assay was performed, the cells were incubated with alamar blue-containing medium according to the manufacturer's protocol (Invitrogen, Cat no. DAL1025), followed by PBS washing and assessment of cytotoxicity using the Sulforhodamine B based assay protocol as described above.

### 2.5 Statistics

Statistical *t*-test calculations were done using Microsoft excel or the R studios program.

## 3 Results

### 3.1 E4orf4-induced cell death in the cancer cell lines utilized in this study

**Fig. 1:**
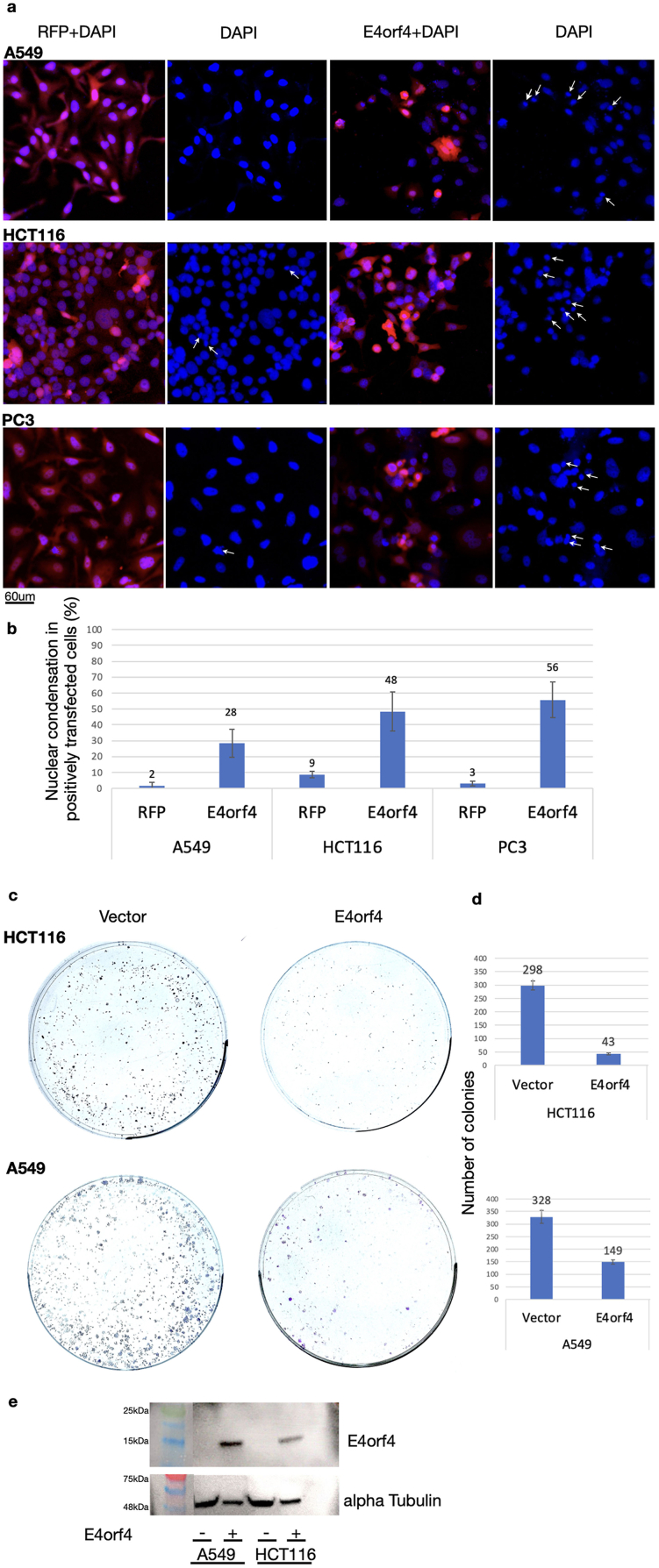
E4orf4-induced cell death in cancer cell lines (a) The indicated cells (A549, HCT116, and PC3) were transfected with plasmids expressing RFP or E4orf4 and were fixed 48 h later. E4orf4-expressing cells were stained with E4orf4-specific antibodies and all cells were counterstained with DAPI. Representative images with merged staining of RFP or E4orf4 together with DAPI, or DAPI staining alone are shown. The arrows point to condensed nuclei. (b) The graphs show the average percentage of transfected cells with condensed nuclei from two independent experiments. Error bars represent standard deviation. (c) Cells transfected with an empty vector or with an E4orf4-expressing plasmid, both containing a zeocin selection marker, were selected with zeocin for 10 days and emerging colonies were stained. (d) The graphs show the average number of colonies from two independent experiments. Error bars represent standard deviation. Only colonies of at least 0.5 mm in diameter were scored. (e) A representative Western blot from the experiments in c and d is shown.

This work seeks to test our hypothesis that E4orf4-induced cancer-specific cell death can be replicated through a combination of drugs that individually target pathways disrupted by E4orf4. We first validated that the cancer cell lines employed as models to assess this hypothesis were indeed susceptible to killing by E4orf4. These cells included HCT116 cells derived from colon cancer, A549 cells originating from lung cancer, PC3 cells obtained from prostate cancer, and MDA-MB-231 cells derived from a triple negative breast cancer. MDA-MB-231 cells have been previously reported to undergo E4orf4-induced cell death [44], and Fig. 1 demonstrates that the other three cancer cell lines are also vulnerable to E4orf4-induced toxicity.

Levels of E4orf4-induced cell death are usually measured by a DAPI assay that quantifies nuclear condensation or other nuclear aberrations rather than by standard apoptotic assays, because E4orf4 does not induce manifestations of classical apoptosis in various cell lines [27,45,46]. Fig. 1a displays representative images of control cells expressing RFP and E4orf4-expressing cells counterstained with DAPI to visualize nuclei, and Fig. 1b exhibits graphs summarizing the quantitation of transfected cells manifesting nuclear condensation. These figures validate previous findings demonstrating that the prevalence of nuclear condensation in the presence of E4orf4 increased significantly in all cancer cell types tested [37,44].

In addition, a clonogenic assay was utilized to assess long-term survival of control and E4orf4-expressing HCT116 or A549 cells. These cells were transfected with an empty vector or a plasmid expressing E4orf4, both containing a zeocin selection marker. Fig. 1c shows representative wells containing colonies derived from the transfected cells, which survived a 10-days zeocin selection in the presence or absence of E4orf4, and Fig. 1d exhibits graphs summarizing two experiments. E4orf4 expression in the cells was verified by Western blot analysis (Fig. 1e). These results demonstrate that E4orf4 expression led to a drastic reduction in colony formation in the two cancer cell lines tested.

Thus, the cumulative results establish that all cancer cell lines tested here are sensitive to E4orf4-induced cell killing and are therefore suitable for investigating cell death induced by E4orf4 mimicry.

### 3.2 Determination of sublethal concentrations of individual inhibitors

In preparation for testing the efficacy of E4orf4-mimicking drug combinations for the elimination of cancer cells with minimal toxicity to normal cells, we determined the maximal drug concentrations that were sublethal when applied individually. The drugs were tested in the cancer cell lines described above, and the immortalized human keratinocyte cell line, HaCaT, served as a normal control.

**Fig. 2:**
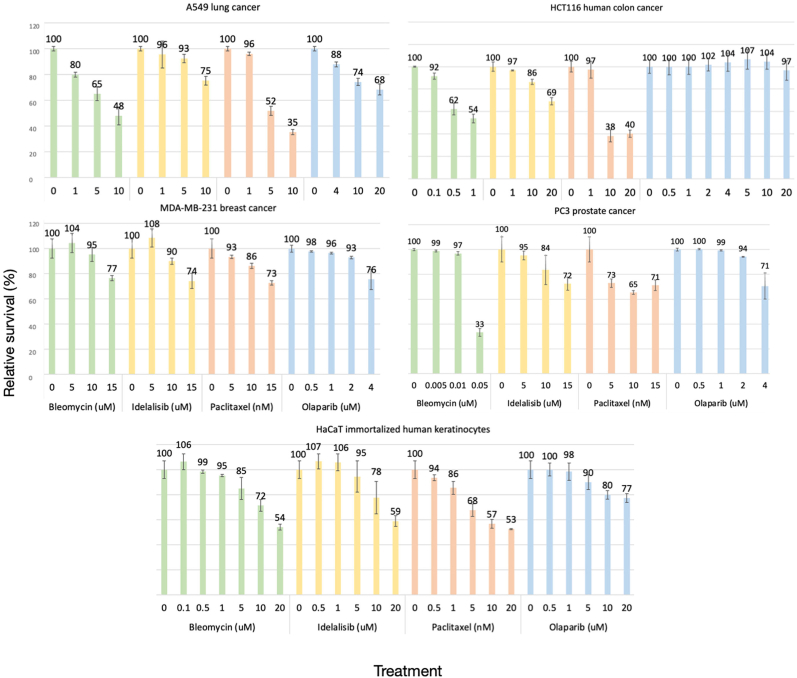
Determination of sublethal concentrations of individual drugs The cells, as indicated in the figure, were subjected to increasing concentrations of the drugs and toxicity was measured. The various drugs are depicted in different colors. The numbers above the graphs indicate the percentage of survival, and survival of untreated cells was defined as 100 %. N > 4, error bars represent standard deviation.

The three drugs used to mimic the known ability of E4orf4 to disrupt cellular pathways regulating the DDR, the cell cycle, and PI3K signaling were described extensively in the literature. They were chosen because their impact on their target pathways and on the cell was similar to the identified E4orf4 effects described above. They include the following FDA-approved drugs: olaparib, a well-known PARP inhibitor [47]; paclitaxel, an inhibitor of microtubule dynamics, which was demonstrated to cause a G2/M arrest and apoptosis, similar to E4orf4 [48]; and idelalisib, a recognized PI3K inhibitor [49]. The fourth drug utilized here, bleomycin, is a DNA breaks inducer used to enhance the stress on DDR pathways [50]. The addition of bleomycin was expected to amplify the cytotoxic effects of DDR inhibition. The cancer cells as well as the normal, non-transformed HaCaT cells were subjected to increasing drug concentrations to determine the concentrations causing less than a 10 % drop in cell survival. Cell survival was assessed using the sulforhodamine B assay, which evaluates protein mass as a measure of cell number [51]. Fig. 2 demonstrates that the non-toxic single drug concentrations were cell line-dependent with slight sensitivity differences between the different cells. Table 1 summarizes the final concentrations chosen for drug combination treatments. For all drugs, none of the sublethal concentrations showed significant toxicity during a prolonged treatment of 3.5 days.

**Table 1:** Final working concentrations of drugs for each cell line.

Cell line	Olaparib (uM)	Idelalisib (uM)	Paclitaxel (nM)	Bleomycin (uM)
HCT116	4	3	1	0.1
A549	4	3	1	0.5
MDA-MB-231	0.5	3	0.5	1
PC3	0.1	1	1	0.01
HaCaT	1	1	1	0.5

### 3.3 Combinations of sublethal concentrations of E4orf4-mimicking drugs dramatically increased toxicity in cancer cell lines

**Fig. 3:**
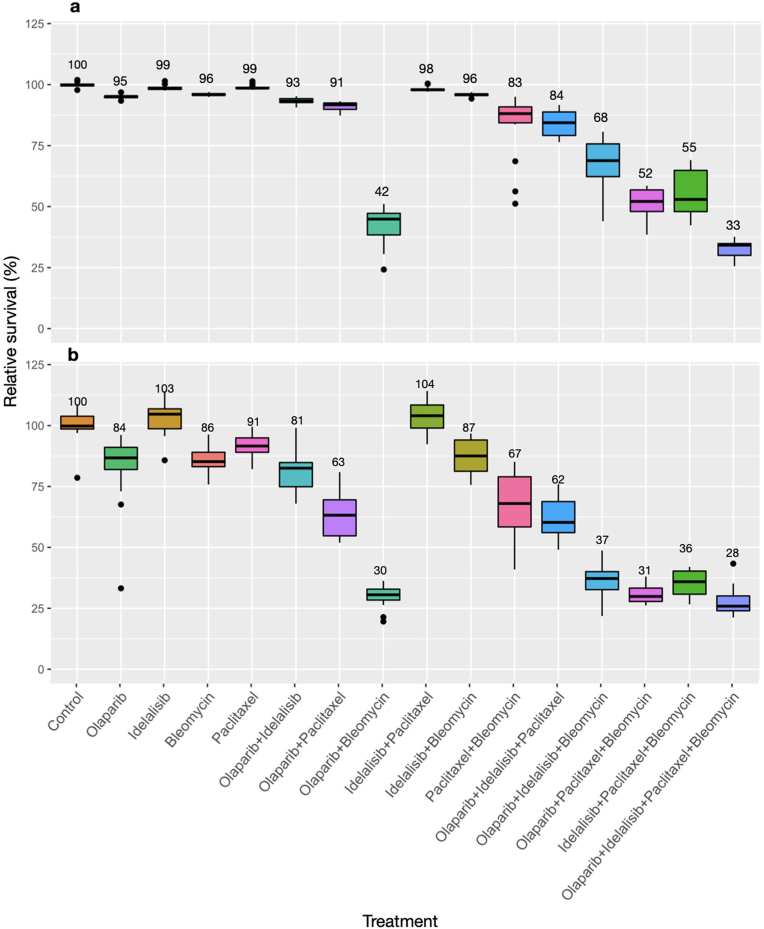
Viability assessment of HCT116 cells following a 3.5-day treatment with E4orf4-emulating drugs (a) Results of a cytotoxicity sulforhodamine B assay. (b) Results of an alamar blue reduction potential assay. Data labels show mean percent viability per treatment. N = 16, error bars represent standard deviation.

Using the single sublethal drug concentrations determined above, which caused little to no change in cell growth compared to control untreated cells, we examined the effects of dual, triple and quadruple drug combinations on HCT116 cells by employing the sulforhodamine B assay. Fig. 3a demonstrates that most dual drug treatments did not greatly increase cytotoxicity in HCT116 cells, with two exceptions. The combination of a PARP inhibitor with bleomycin, an inducer of DNA breaks, resulted in a dramatic decrease in cell viability, reaching only 42 % cell survival. A much smaller effect was observed upon treatment of the cells with paclitaxel and bleomycin, resulting in 83 % survival (p < 0.0005). The examination of the outcomes of treatments with four different triple drug combinations revealed a large variability in the responses, ranging between 52 % and 84 % survival. Interestingly, the addition of a third drug to the dual drug combinations did not always result in an increased effect. While the combination of olaparib and bleomycin caused a significant cell mortality (42 % survival relative to control, p < 0.0005)), the further addition of either idelalisib or paclitaxel reduced the effect by 26 % (p < 0.0005) and 9 % (p < 0.0005), resulting in 68 % and 52 % survival, respectively. However, the combination of all four drugs at sublethal individual doses resulted in a substantially increased toxicity with reduction of cell viability to a mere 33 % relative to control (p < 0.0005), and was still statistically more lethal than the second most potent combination of just olaparib and bleomycin (p < 0.0005).

A second assay designed to estimate the drug effects on cell viability, the alamar blue assay, was also used. Alamar blue is an oxidation-reduction indicator, which detects the levels of oxidation during respiration, and is non-toxic to the cells, thus serving as a gauge of cell health [52]. Fig. 3b demonstrates that the alamar blue assay was more sensitive in identifying single-dose drug-induced changes in HCT116 cells. Furthermore, in contrast with the sulforhodamine B assay, the alamar blue assay demonstrated a more profound effect of triple drug combinations that included bleomycin. These combinations caused a decrease in cellular reduction potential of an average of 66 %, to 34 % of control untreated cells, and the addition of the fourth drug, while statistically significant (p < 0.005) had a modest additional effect. It also had a modest effect compared with the dual combination of the PARP inhibitor olaparib and bleomycin (p < 0.0005). It is likely that there is a threshold effect on respiration such that effects below it do not lead to cell mortality.

**Fig. 4:**
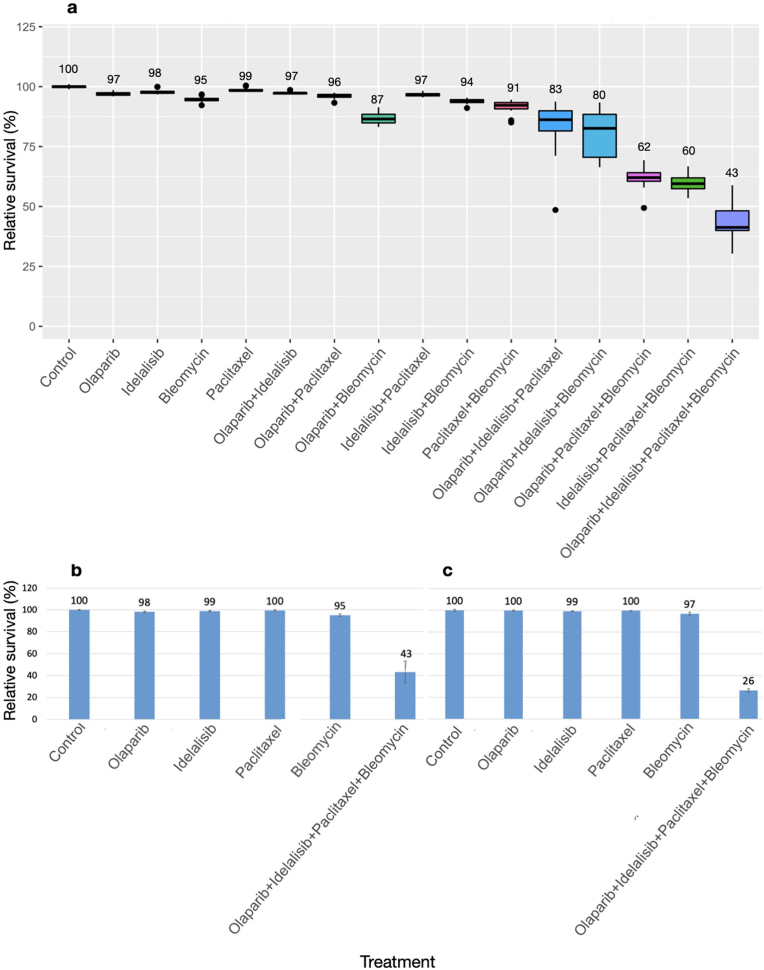
Viability assessment of A549, MDA-MB-231 and PC3 cells following a 3.5-day treatment with E4orf4-emulating drugs Cytotoxicity assay results in A549 lung cancer cells (a), MDA-MB-231 breast cancer cells (b), and PC3 prostate cancer cells (c) after a 3.5-day drug treatment. Data labels show mean percent viability. N > 3, error bars represent standard deviation.

Similar results were obtained in the A549 lung cancer cells (Fig. 4a). These cells were less sensitive to the olaparib-bleomycin dual combination, which caused only a mild 13 % loss in cell survival to 87 % of control. All other dual combinations were even less effective. Noticeably, the combination of three drugs in these cells had a greater potency when paclitaxel and bleomycin were included together. As observed in HCT116 cells, the strongest toxic effect was detected when all four drugs were combined together, with an increase of cellular toxicity of roughly 60 % in comparison to control (p < 0.0005), resulting in 43 % survival. The added value of a four-drug combination vs. three drugs was somewhat greater in HCT116 cells compared with A549 cells, further reducing cell survival to 63.5 % vs 71.7 % of the most potent triple drug combination respectively.

**Fig. 5:**
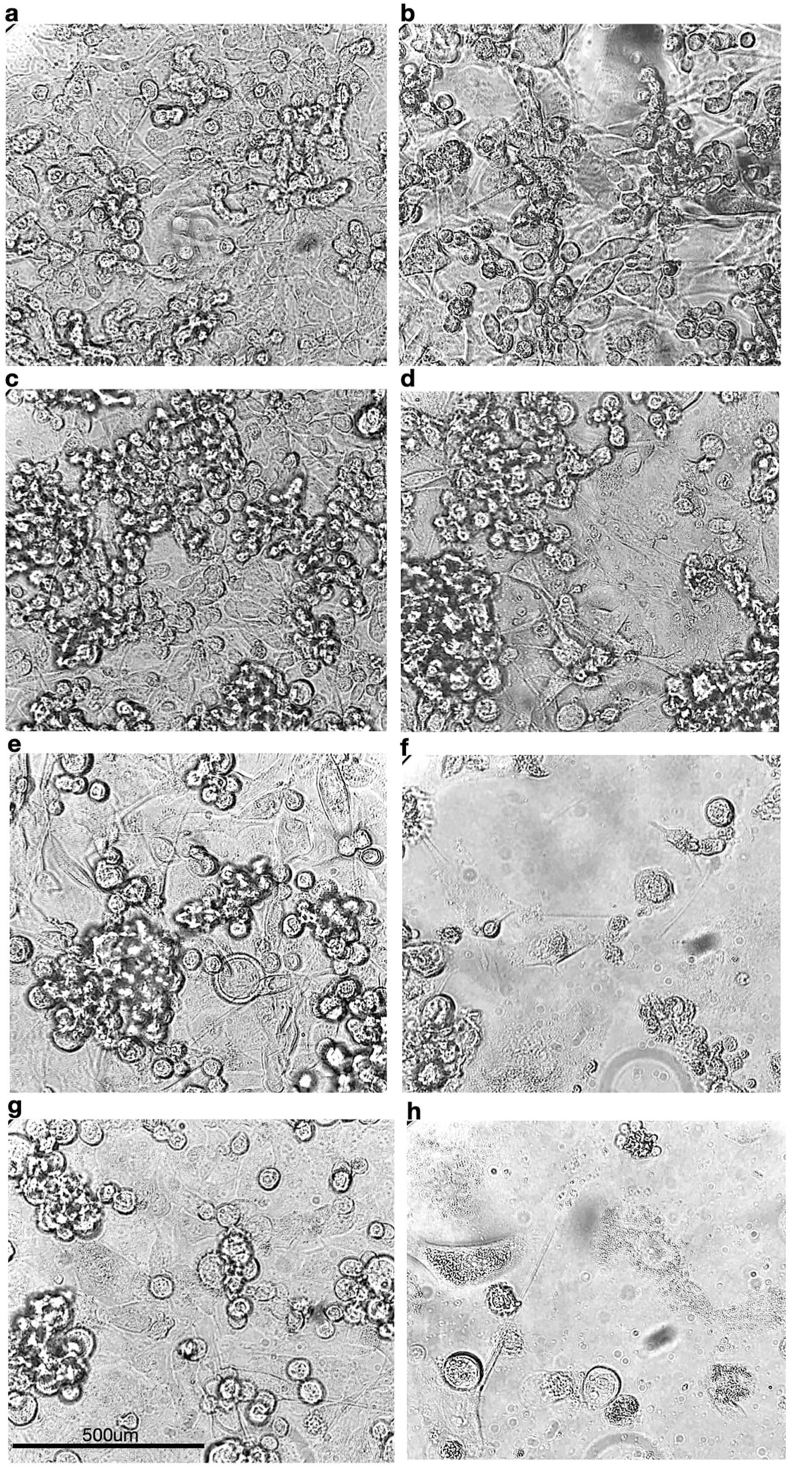
Microscopic images of MDA-MB-231 and PC3 cells after a 3.5-day drug treatment Before fixation and cytotoxicity analysis, cells were photographed using bright field light microscopy at 100× magnification. MDA-MB-231 control cells (a) and cells treated with olaparib (b), idelalisib (c), bleomycin (d), paclitaxel (e) and a combination of all four drugs (f) are presented here for comparison. PC3 control cells and cells treated with the 4-drug combination are shown in (g) and (h) respectively. The bar at the bottom on the left represents 500 μM.

Experiments comparing the individual drug treatments with the quadruple drug combination were performed in MDA-MB-231 breast cancer cells and in PC3 prostate cancer cells. The results revealed over 55 % and 70 % increase in cellular toxicity induced by the quadruple drug combination respectively (p < 0.0005) (Fig. 4b and c). Cells that remained attached to the wells after a 3.5-day treatment appeared swollen, rounded and morphologically aberrant (Fig. 5).

### 3.4 The tested drug combinations did not induce enhanced toxicity in non-cancerous cells

**Fig. 6:**
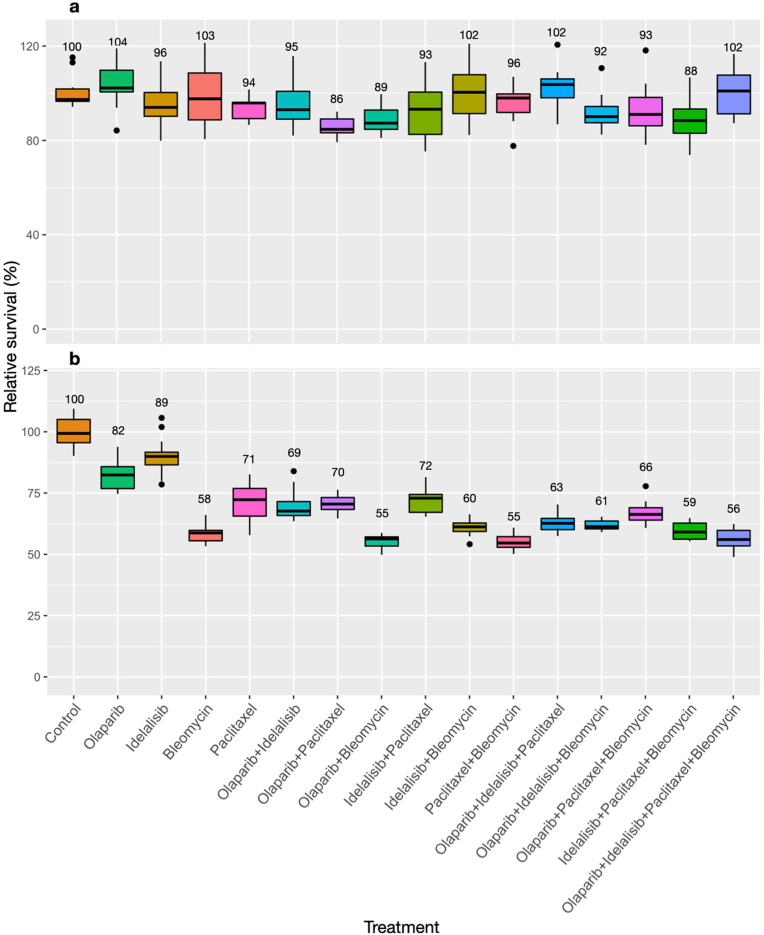
The combination of sublethal concentrations of the drugs did not act synergistically in the non-cancerous HaCaT cells The non-cancerous HaCaT cells were treated and analyzed as described in the legends to Fig. 3, Fig. 4, using the appropriate sublethal drug concentrations. (a) Results of the sulforhodamine B assay. (b) Results of an alamar blue assay. N = 16, error bars represent standard deviation.

In contrast to the results described above, combinations of sublethal levels of E4orf4-emulating drugs did not cause a significant cellular cytotoxicity in the non-cancerous HaCaT cell line, and cell proliferation was not decreased (Fig. 6). Specifically, both the cytotoxicity and the alamar blue assays demonstrated that even the quadruple drug combination did not increase the effect of a single treatment with bleomycin in HaCaT cells. These results indicate that the single drugs did not act in synergy within the drug cocktail in these cells.

## 4 Discussion

The use of E4orf4 as a guide for targeting specific cellular pathways in order to imitate its anti-cancer properties holds a promising potential for further investigation. While the single treatments at sublethal concentrations showed almost no effect in several cancer cell lines, their combination exhibited profound changes in cell viability similar to E4orf4's effects reported in the past and in this work. The response of two different cancer cell lines, HCT116 and A549, to double and triple E4orf4-like drug combinations was not identical. For example, the combination of the PARP inhibitor olaparib and the DNA breaks-inducer bleomycin was much more toxic in HCT116 cells, and addition of idelalisib or paclitaxel to this combination increased its toxicity in A549 cells but decreased its toxicity in HCT116 cells. Thus, various types of cancers may respond differently to partial E4orf4 mimicry. However, when applying a quadruple E4orf4-mimicking drug cocktail, both cell lines responded significantly better, manifesting increased toxicity. Interestingly, the degree of toxicity induced by the quadruple drug combination in PC3, HCT116, and A549 cells (26 %, 33 %, and 43 % cell survival, respectively) mirrored their varied sensitivity to E4orf4-induced cell death (56 %, 48 %, and 28 % cell death, respectively, as measured by the DAPI assay).

Our results suggested that the combined, additive toxicities of the single drug treatments in the four different cancer cell lines were in the range of 4–11 %. However, the actual toxicities induced by the four-drug combination ranged between 57 % and 74 %. These results are strongly consistent with the suggestion that the four drugs acted in synergy in these cancer cells, generating dramatically enhanced toxicity compared with the single drugs. Similarly, when comparing the triple and quadruple drug combinations, the toxicity of the quadruple combination was higher than the sum of the triple combinations and the fourth single drug. Drug synergy can be further investigated by several mathematical models described in the literature, which analyze the effects of combinations of a range of concentrations of the drugs under study [53]. In contrast to the cancer cell lines, no sign of synergy emerged from parallel experiments in the non-cancerous HaCaT cell line.

Disruption of the diverse E4orf4 target pathways will likely have a varied degree of contribution to the elimination of different types of cancer, depending on the nature of the genetic and epigenetic alterations in these cells. Therefore, the more accurate the E4orf4 emulation is, the more effective it will be in a greater variety of cancers, thus imitating the ability of E4orf4 to kill a large number of cancer cell types [18,38]. Nonetheless, the effective concentrations of the constituents of the E4orf4-like drug cocktail will conceivably have to be adjusted separately for each type of cancer, as seen here.

E4orf4 associates with numerous cellular proteins participating in several cellular pathways [18,19]. Thus, to increase the efficacy of E4orf4-mimicking drug cocktails, the continued study of E4orf4 interactions with cellular pathways is important, as it is likely to suggest the inclusion of more drugs that target novel pathways to improve the mimicry of E4orf4. Additional potential E4orf4-mimicking drugs will also serve to replace other drugs in the cocktail if resistance to these pharmaceuticals has emerged. This is reminiscent of the use of drug cocktails against HIV infection, which include several drugs targeting numerous mechanisms underlying HIV replication [54].

Furthermore, because the E4orf4-like individual drugs are applied at sublethal concentrations, it may be possible to utilize additional drugs targeting the same pathways, which cannot currently be employed for monotherapy due to their high toxicity. The feasibility of inclusion of such drugs within the cocktail will increase the arsenal of drugs that can be used according to the E4orf4 guide.

The sulforhodamine B cytotoxicity and the alamar blue assays manifested different sensitivity levels, with the metabolism-based alamar blue assay being more sensitive in detecting effects of single drugs as well as of drug combinations. Metabolic alterations often precede changes in morphology and broad protein expression levels, possibly explaining why the sulforhodamine B cytotoxicity assay, which is based on total protein quantification, detected a decreased effect of some treatments in comparison to measurements using alamar blue. It is conceivable that given enough time, differences between the two methods would close the gap to some extent. On the other hand, one must bear in mind that some metabolic changes can be well tolerated by the cells without significantly reducing proliferation or increasing cytotoxicity. Thus, not all drug-induced changes detected by the alamar blue assay would be detected by the sulforhodamine B cytotoxicity assay even after prolonged periods of time.

The observation that little to no effect was seen in non-cancerous cells treated with the drug combinations discussed here, underscores the heightened vulnerability of cancer cells to the simultaneous disruption of various regulatory pathways. Cancer cells are often dependent on multiple oncogenes that drive key signaling pathways [55]. Interfering with these pathways - many of which already harbor cancer related mutations - can lead to cell death. For example, blocking DDR pathways could be particularly effective, as many cancer cells have compromised DNA damage signaling and rely heavily on the residual DDR for survival. Similarly, targeting the cell cycle has a greater impact on rapidly dividing cancer cells. Since DNA Tumour viruses often target the same cellular pathways to ensure efficient replication, studying virus-induced changes in cellular regulation has proven highly relevant to cancer research [[56], [57], [58], [59]].

The research presented here serves as a proof-of-principle study, laying the groundwork for further exploration of the impact of E4orf4-like drug cocktails in tissue-based and *in vivo* models. Assessing the *in vivo* efficacy and organ toxicity of these new treatment combinations, along with drug-drug interactions, pharmacokinetics, and pharmacodynamics, will be essential for their progression into common medical practice.

In conclusion, while studies of viral-host interactions are critical for understanding viral biology and pathogenicity, this research can also offer valuable insights for addressing diseases like cancer. Discoveries in virus biology have already led to the development of oncolytic viruses for cancer therapy. Our research described here provides another approach for applying virus biology to enhance cancer treatment, using the viral E4orf4 protein as a guide for novel cancer therapies in the form of E4orf4-mimicking drug cocktails. The use of this E4orf4-based strategy circumvents the need for the prolonged and challenging development of new drugs and allows for the low-toxicity and effective use of existing drugs at sublethal concentrations. The insights gained in this work could lead to the development of treatments that are both effective and readily implementable, ultimately enhancing patient outcomes and moving cancer treatment forward.

## CRediT authorship contribution statement

**Amir Basis:** Writing – original draft, Visualization, Investigation, Formal analysis, Conceptualization. **Rakefet Sharf:** Project administration, Investigation, Formal analysis. **Tamar Kleinberger:** Writing – review & editing, Writing – original draft, Supervision, Funding acquisition, Conceptualization.

## Declaration of generative AI and AI-assisted technologies in the writing process

During the preparation of this work, the authors used ChatGPT in order to improve the English. After using this tool, the authors reviewed and edited the content as needed and take full responsibility for the content of the publication.

## Declaration of competing interest

The authors declare that they have no known competing financial interests or personal relationships that could have appeared to influence the work reported in this paper. Funding sources were not involved in the conduct, analysis, or submission of this research.

## Data Availability

The data is included in the paper.
